# Migratory patterns and settlement areas revealed by remote sensing in an endangered intra-African migrant, the Black Harrier (*Circus maurus*)

**DOI:** 10.1371/journal.pone.0210756

**Published:** 2019-01-17

**Authors:** Marie-Sophie Garcia-Heras, Beatriz Arroyo, François Mougeot, Keith Bildstein, Jean-François Therrien, Robert E. Simmons

**Affiliations:** 1 Department of Forest Ecosystems and Society, Oregon State University, Corvallis, United States of America; 2 FitzPatrick Institute of African Ornithology, DST-NRF Centre of Excellence, University of Cape Town, South Africa; 3 Instituto de Investigación en Recursos Cinegéticos (IREC), CSIC-UCLM-JCCM, Ciudad Real, Spain; 4 Hawk Mountain Sanctuary, Acopian Center for Conservation Learning, Orwigsburg, United States of America; University of Lleida, SPAIN

## Abstract

Annual movements have been widely described for birds migrating across the Americas and between Eurasia and Africa, yet relatively little information exists for intra-African migrants. Identifying the areas used throughout a species annual cycle by understanding migratory patterns and settlement areas during breeding and non-breeding seasons is essential for conservation initiatives. Here, we describe for the first time, the migratory patterns and settlement areas of an endangered raptor endemic to Southern Africa, the Black Harrier (*Circus maurus*). From 2008 to 2015, thirteen breeding adult Black Harriers were trapped in south-western South Africa and fitted either with a GPS-GSM or with a PTT tracker device. Adults were monitored for 365 ± 198 days (range: 56–819 days) revealing great individual variability in annual movements. Most Black Harriers performed an unusual West-East migration from their breeding areas, but routes of all migrating individuals covered the entire southern land area of South Africa and Lesotho. The distance travelled averaged 814 ± 324 km, but unlike many other species, migrants travelled faster during post-breeding (i.e. austral summer) (207.8 ± 113.2 km.day^-1^) than during pre-breeding (i.e. austral winter/spring) migrations (143.8 ± 32.2 km.day^-1^). Although most marked individuals displayed movements similar to those that bred following pre-breeding migrations, only two of thirteen were confirmed as breeders the year after being tagged. This suggests that individuals may sometimes take a sabbatical year in reproduction, although this requires confirmation. Most tagged birds died on migration or during the non-breeding season. Adults frequently returned to the same non-breeding settlement areas, and often used up to 3 different locations an average of about 200 km apart. On the other hand, there was wide variation in distance between subsequent reproductive events. We discuss the implications of our study for the conservation of Black Harriers and more broadly for intra-African bird migrants.

## Introduction

Understanding the conservation requirements of threatened species requires consideration of their entire annual cycle. The non-breeding period, including migrations to and from non-breeding areas for migratory species, is a critical time for many animals, during which individuals may face harsh environmental conditions or reduced resource availability ultimately affecting their survival [1, 2]. Paradoxically, the vast majority of the studies investigating the ecology of species focus on their breeding seasons despite the fact that migrants spend the majority of their annual cycle *en route* or in their non-breeding areas [2, 3], a period when mortality is frequent [4]. Additionally, conditions encountered outside the breeding area may affect reproduction through carry-over effects on body condition and arrival date back to the breeding grounds [5, 6]. Identifying areas used throughout the annual cycle is therefore critical as this helps identify limiting factors outside the breeding areas, which may be essential for understanding population processes [2, 7–10].

To date, most studies of bird migration have been performed on intercontinental migrants, i.e. individuals traveling thousands of kilometers from their breeding to their non-breeding areas [11–14], while species travelling shorter distances, without necessarily moving across country borders have received, by comparison, relatively less attention [14–16]. Among raptor species, investigations have so far focused on species migrating across the Americas (e.g. [17]), and between Eurasia and Africa (e.g. [2, 13, 18]), whereas little is known about intra-African migrants [19, 20]. In a context where many African raptor populations have decreased in recent decades [21, 22], mostly due to habitat fragmentation, persecution [23] and exposure to poisons and contaminants (e.g. [24, 25]), there is an urgent need to learn more about migration routes and areas used by African raptor species throughout their annual cycle, to effectively manage and conserve these threatened populations.

The Black Harrier (*Circus maurus*) is a medium-sized bird of prey endemic to southern Africa. Its global population has been estimated at less than 1,000 breeding individuals, and the species is currently considered endangered in South Africa, Namibia and Lesotho [26, 27]. This ground-nesting raptor only breeds in indigenous vegetation, essentially along the south-western South African coast [28–30]. Comparisons of Black Harrier reporting rates and areas occupied from the South African Bird Atlas (SABAP) project 1 (1987–1991) and SABAP project 2 (2007- present) suggest that the population has declined over the last 4 decades [31, 32]. The loss and fragmentation of Black Harrier’s natural breeding habitats has been suggested as one of the main factors explaining the current scarcity of the species [28, 30], but equally, this negative trend may also be linked to effects occurring within the species’ non-breeding areas, including its migration routes. Whereas our understanding of Black Harriers’ genetics, breeding ecology and health has been advanced in recent years [33–36], published information on Black Harrier migration, movement patterns and settlement areas during the breeding and non-breeding season is lacking. This is important given that recent studies have shown that exposure to organochlorine compounds during Black Harrier’s breeding season may have adverse effects on indicators of health [24, 30], and this exposure may have occurred during the non-breeding seasons. Although broad-scale areas of presence and absence have been identified by SABAP 1 and 2 surveys during the non-breeding season, no information based on individually marked birds yet exists.

Using location data collected from adult Black Harriers marked with GPS-GSM or PTT tracker devices and monitored during the 2008–2016 breeding and non-breeding seasons, the aims of this study were to (i) describe for the first time the overall migration patterns (i.e. routes, distance, daily speed), (ii) identify the areas used during the breeding and non-breeding seasons and (iii) estimate the size of the settlement areas used by individuals during both the breeding and the non-breeding seasons. We discuss the implications of our findings for the sustainable conservation of Black Harriers in Southern Africa, and for intra-African migrants more broadly.

## Materials and methods

### Study area, capture and marking

Field work took place in two main regions in South Africa within Black Harrier´s breeding range: (i) along the coast of the Western Cape Province (hereafter Western Cape), in the Cape Floral Kingdom (or Fynbos biome) in an area north of the city of Cape Town (33.700° S, 18.450° E; 33.133° S, 18.083° E), and (ii) inland in the Northern Cape Province (hereafter Northern Cape), in the Karoo biome in the Nieuwoudtville area (31.316° S, 19.083° E; see [30, 34] for additional details). Nests were found as described in [34]. Once nestlings were 15–39 days old, we caught breeding adults using a Dho Gaza net and a stuffed Spotted Eagle Owl (*Bubo africanus*) set at a distance of 20–30 m from active nests. We also trapped a female that failed to respond to the owl decoy using a nest-trap placed over the nestlings. After capture, adult birds were weighed, measured and individually marked with a metal and a colored ring with a unique alpha-numeric code. Nestlings were also ringed similarly prior to fledging for potential re-sightings after dispersal. We discriminated captured adult males from females based on behavior (i.e. adult females and males behave very differently during the breeding season; see [37, 38] for more details), weight and size: Black Harriers exhibit reversed sexual dimorphism, females being about 7% larger than males in wing length, and about 28% heavier than males; [30, 38], with no overlap in weight (see below for captured individuals). Each bird was fitted with either a satellite (9.5 g solar-powered Platform Transmitter Terminal, PTT-100, Microwave Telemetry inc., Maryland, USA) or a GPS-GSM (16 g solar-powered GPS-GSM SAKER-UL Tracker, Ecotone Telemetry, Poland) transmitter. In all cases, the transmitters were attached with backpack harnesses made of Teflon [39]. The average mass of the birds was 493.3 ± 37.2 g (range: 450–580 g, n = 9) for females and 396.3 ± 11.1 g (range: 380–405 g, n = 4) for males. Therefore, a GPS-GSM transmitter and harness relative mass was about 4% and 3% of the males’ and females’ mass, respectively, and a PPT transmitter was about 2% for both sexes. As previously described in similar studies on Montagu’s (*C*. *pygargus*) and Pallid (*C*. *macrourus*) Harrier’s [4, 13, 40], there was no evidence that this additional weight had detrimental effects on the tagged Black Harriers. All birds were released unharmed at their capture site within 20 min. None abandoned their nest following capture, and all birds quickly resumed food provisioning of their nestlings after tagging. Fieldwork protocols were approved by the University of Cape Town’s science Faculty Animal Ethics Committee (Permit number: A1/2014/2013/V21/GC), the Cape Nature permit from the Western Cape Province (number AAA007-00099-0056), and the Northern Cape Province- Fauna permit (FAUNA 1120/2013, ODB Number 2862/2013).

Location data from PPT transmitters were collected using the Argos satellite system, which also provides a nominal location accuracy [41]. Location class (“LC”) 0, 1, 2, and 3 indicate that the reported location follow a normal distribution with a standard deviation of > 1 km, ≤ 1 km, ≤ 350 m and ≤ 150 m of the true location, respectively [42]. By contrast, location classes A, B, C and Z are not associated with reported accuracy and are less reliable. Thus, only classes 1, 2, and 3 were selected for our analyses. When for a same location and time, two classes were available, only the highest accuracy class was selected for analyses. All PTTs were programmed to record one location every hour every day, although on average locations were received every other day. However, some devices occasionally recorded locations at a shorter time interval within a single day. Thus, in order to standardize our data and to avoid potential biases associated with the non-independence of the data, positions obtained less than 30 min apart were excluded from analyses [43]. In the case of the GPS-GSM trackers, the accuracy of the locations recorded was ≤ 5 m. Locations were recorded daily, every 30 min from 0500 to 2000, the GPS locations being stored and transmitted when in GSM coverage. A few locations coming from PPT devices were defined as aberrant (i.e. in the ocean, n = 17) which we excluded from all data bases.

A total of 13 breeding birds (9 females and 4 males), among which 6 were pairs, were equipped throughout July-December 2008–2015 breeding seasons. Seven birds were fitted with PTT (n = 2 in 2008, n = 1 in 2010, n = 1 in 2012, and n = 3 in 2013) and six with GPS-GSM tracker devices (n = 3 in 2013, n = 2 in 2014, and n = 1 in 2015) (S1 Table). Marked birds were monitored for an average of 365 ± 198 days (range: 56–819 days), with a total of 4545 locations selected for analyses, all birds combined (range: 111–1254 locations per individual). Overall, the amount of data collected varied among birds due to mortality, device failures or discharged batteries (e.g. lower access to sunlight after consecutive days of cloudy or rainy skies, or during incubation when the females’ scapular feathers may cover the solar panel preventing exposure to sunlight). Some individuals were therefore monitored for only one migration event (n = 4 birds for post-breeding migrations) and others for longer (n = 9; see S1 Table for more details). Most transmitters (n = 10) stopped emitting for unknown reasons, and we confirmed the death of 3 birds by locating their carcasses in the field (details below). The last tagged bird was monitored until January 2016.

Prior to all analyses, all locations were adequately projected to the Universal Transverse Mercator coordinate system, i.e. WGS 84-UTM Zone 34S or WGS84-UTM Zone 35S, depending of their geographical position.

### Migration patterns

We defined two types of migration events: (i) post-breeding migration (typically occurring in the austral summer in December-January) was defined as all recorded movements occurring after an individual departed from its breeding area until it reached its first non-breeding settlement area (see below), or (ii) pre-breeding migration (usually occurring in the austral winter/spring in July-August) was defined as all recorded movements occurring after an individual departed from its last non-breeding settlement area to return to its breeding area. The beginning of a migration event was obvious, as movements were initiated with more than 100 km travelled within a day. Likewise, the end of a migration event was also obvious, as individual’s daily movements became shorter than 35 km day^-1^. After migrations, tracked birds usually clustered around a given place (hereafter settlement area) or conducted a prospecting-like behavior (see results below) at the beginning of the breeding season. In most cases, we obtained locations during the exact day the bird left for or arrived from a migration event. However, in a few cases, the departure or arrival date occurred between two locations that were more than 24 h apart. In these cases, we estimated the departure or arrival date as the mean dates between the two locations (rounded up if the mean was not an exact figure).

The distance travelled during a migration event was calculated by summing the total number of kilometers between successive locations until the migration event was completed. Similarly, we calculated the average daily speed (km.day^-1^) by dividing the distance travelled by the migrating bird by the number of days needed to complete a migratory event. All data are presented as means ± SD.

For the purpose of this work, we defined three different types of migratory birds depending on the distances travelled during a migration event: (i) long-distance migrants as those individuals that travelled more than 400 km from their nesting or breeding areas to their non-breeding areas; (ii) median-distance migrants as those individuals that travelled 100–400 km from their nesting or breeding areas to their non-breeding areas and; (iii) short-distance migrants/residents as those individuals that travelled less than 100 km from their nesting area, or that never left it (S1 Table). This choice was based on the frequency distribution of migration distances, which suggested those thresholds, coupled with the fact that all birds thus classed as “long-distance migrants” moved outside the Western Cape Province, whereas those classed as “short-distance migrants/residents” and “medium-distance migrants” remained in the Western Cape Province (see results).

Additionally, where available, we also included information about locations of re-sightings of color-ringed individuals to add qualitative information about distance or direction of post-breeding movements, or fidelity to natal areas.

### Movements after pre-breeding migration

At the end of pre-breeding migrations, when adults returned to their typical breeding range [34], we carefully inspected each tagged individuals’ movements plotting the telemetry locations on Google Earth. In most cases, individuals started a pre-breeding prospecting behavior by reducing their daily movements, i.e. lower daily speed and small distances travelled between two locations. Subsequently, when individuals showed a breeding-like behavior, i.e. by reducing and clustering their movements usually to < 15 km from a fixed point, we went on site to identify whether an active nest existed using a combination of visual observations and the location estimates of GPS-GSM or PTT tracker devices. If an active nest (i.e. with eggs, nestlings or fledglings) was found, then that bird was classed as an active breeder at that time. If, however, after 2–3 consecutive days of at least 5 h of observations per day, no active nest was found, and/or no clear breeding behavior was observed (i.e. aerial food exchanges between male and female, aerial displays of male, aggressive behaviors against competitors or threats; [38]), then that individual was presumed to be a non-breeder.

### Home ranges

To delineate the different areas used by Black Harriers during the non-breeding season, we visually inspected the estimated locations of each bird in QGIS 2.18.2 [44] and identified location clusters (i.e. points grouped in space and time), which we categorized as “settlement areas”. Each settlement area was defined as including locations clustered around a centroid during at least 10 consecutive days (i.e. excluding locations of areas that Black Harriers passed through briefly). The maximum distance travelled by a Black Harrier away from the centroid was 20 km within a single day, so clusters of points located further than 20 km were considered as separate settlement areas. The centroid point of each settlement area was calculated using the analysis tool “mean coordinate”. Centroids of different settlement areas for a given individual were more than 40 km apart.

Home range sizes (i.e. surface area of each settlement area, in km^2^) were determined for each identified settlement area used by 1) confirmed breeders, 2) suspected non-breeders, or 3) migrants during the non-breeding season. For birds that did not initiate a post-breeding migration or that stayed longer in their breeding area than the majority of migrants, we used the average departure dates of migratory birds to differentiate breeding settlement areas and non-breeding settlement areas. Here, we removed night-time locations from the data sets to concentrate on possible foraging locations for the calculation of home ranges. We estimated the home range, or utilization distribution (UDs) of individuals, by means of a kernel density approach [45, 46, 47] using the “adehabitatHR” [48, 49] and “rgdal” [50] packages in R 3.4.4 for each settlement area (breeding and non-breeding season) of monitored birds. Home range sizes were estimated from the fixed 90% kernel density contours using the h_ref_ method and a grid of 100 m. For each individual, we also calculated the distances between either the settlement’s centroid (for non-breeding settlement areas and for breeding areas of suspected non-breeders) or the nest (for breeding areas) and its surrounding locations using the package “aspace” in R.

### Statistical analyses

All statistical analyses were conducted using R.3.4.4 [51]. To evaluate whether the duration, distance travelled and average daily speed of migrants during a migration event differed according to sex or migration type (2 level factor: post-breeding vs. pre-breeding), we conducted General Linear Mixed Models (GLMMs, statistical package lme4, [52]), where response variables were fitted to a Gaussian distribution with an identity link function. Models also included individual ID as a random effect to account for the non-independence of data coming from the same individual.

We also tested, with GLMMs using individual ID as a random term, whether the 90% kernel home range sizes (log-transformed) varied in relation to individual stage (3-level factor: confirmed breeders, suspected non-breeders, migrants during the non-breeding season), sex, type of device (2-level factor: GPS-GSM vs. PTT), and the number of daily locations (continuous variable). The interactions between type of device and number of daily locations, the type of device and individual stage, and the sex and individual stage were also tested, and were dropped from the initial models when not significant. Additionally, we tested the same relations separating the data from GPS-GSM and PTT devices to identify potential intra-device differences.

## Results

### Migratory movements

**Fig 1:**
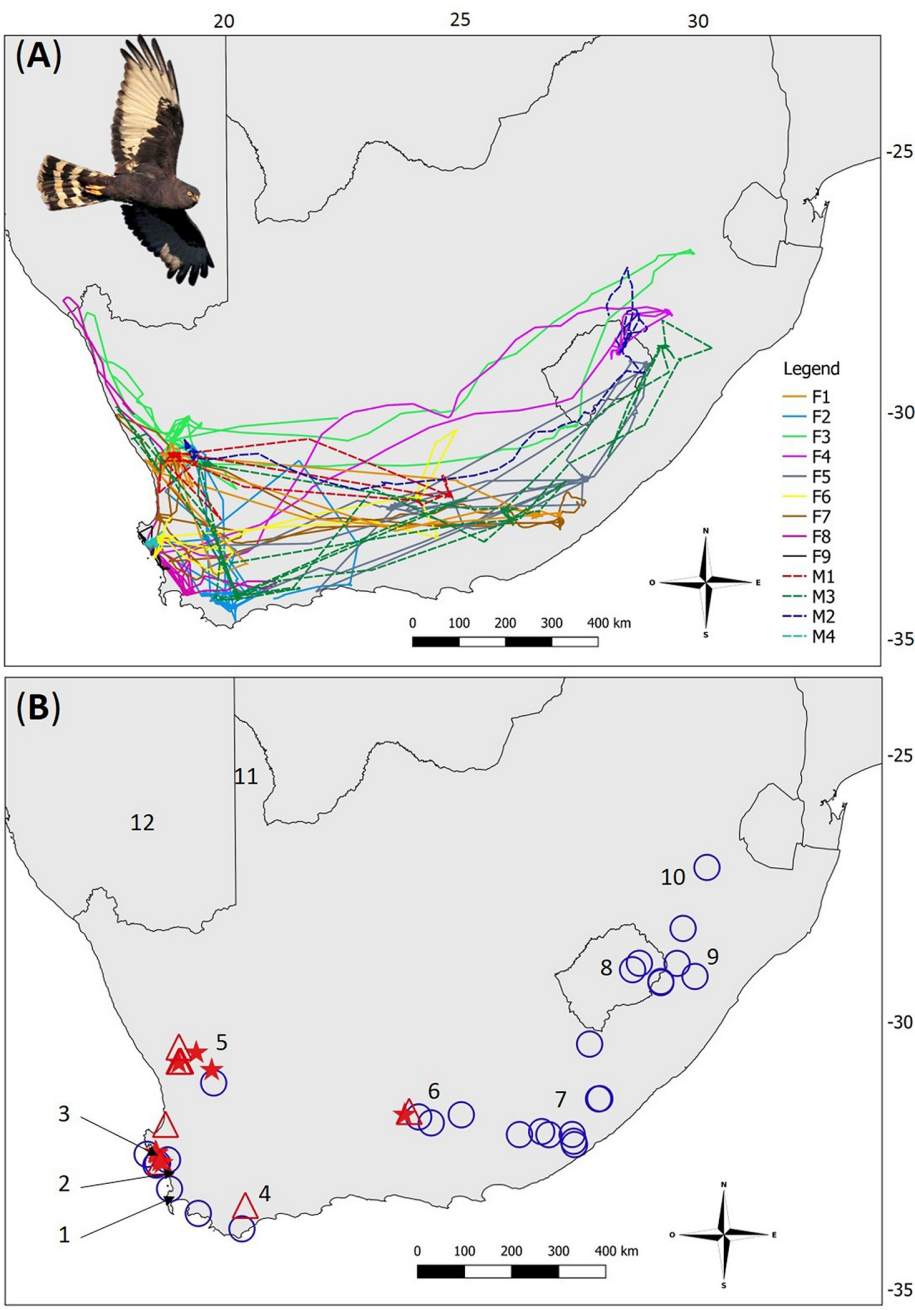
Overall annual movements and identified settlement areas of the 13 marked Black Harriers in Southern Africa. (A) Annual movements of 13 adult Black Harriers marked with GPS-GSM (n = 6) or PTT (n = 7) tracker devices and followed in south-western South Africa during the 2008–2016 period. Females (F1-F9) are shown with colored solid lines, and males (M1-M4) with colored dashed lines. (B) Identified settlement areas used by the 13 adult Black Harriers during the 2008–2016 non-breeding (n = 33, circles with blue contours) and breeding seasons (confirmed nests: n = 15, filled red stars; suspected non-breeders: n = 5, open red triangles). Numbers identify locations of areas mentioned in the text: 1) Cape Town, 2) Koeberg Private Reserve, 3) West Coast National Park, 4) Overberg region, 5) Nieuwoudtville area, 6) Camdeboo Mountains, 7) Stutterheim area, 8) Lesotho, 9) Mooi River area, 10) Daggakraal area, 11) Kgalagadi Transfrontier Park, 12) Namibia.

**Fig 2:**
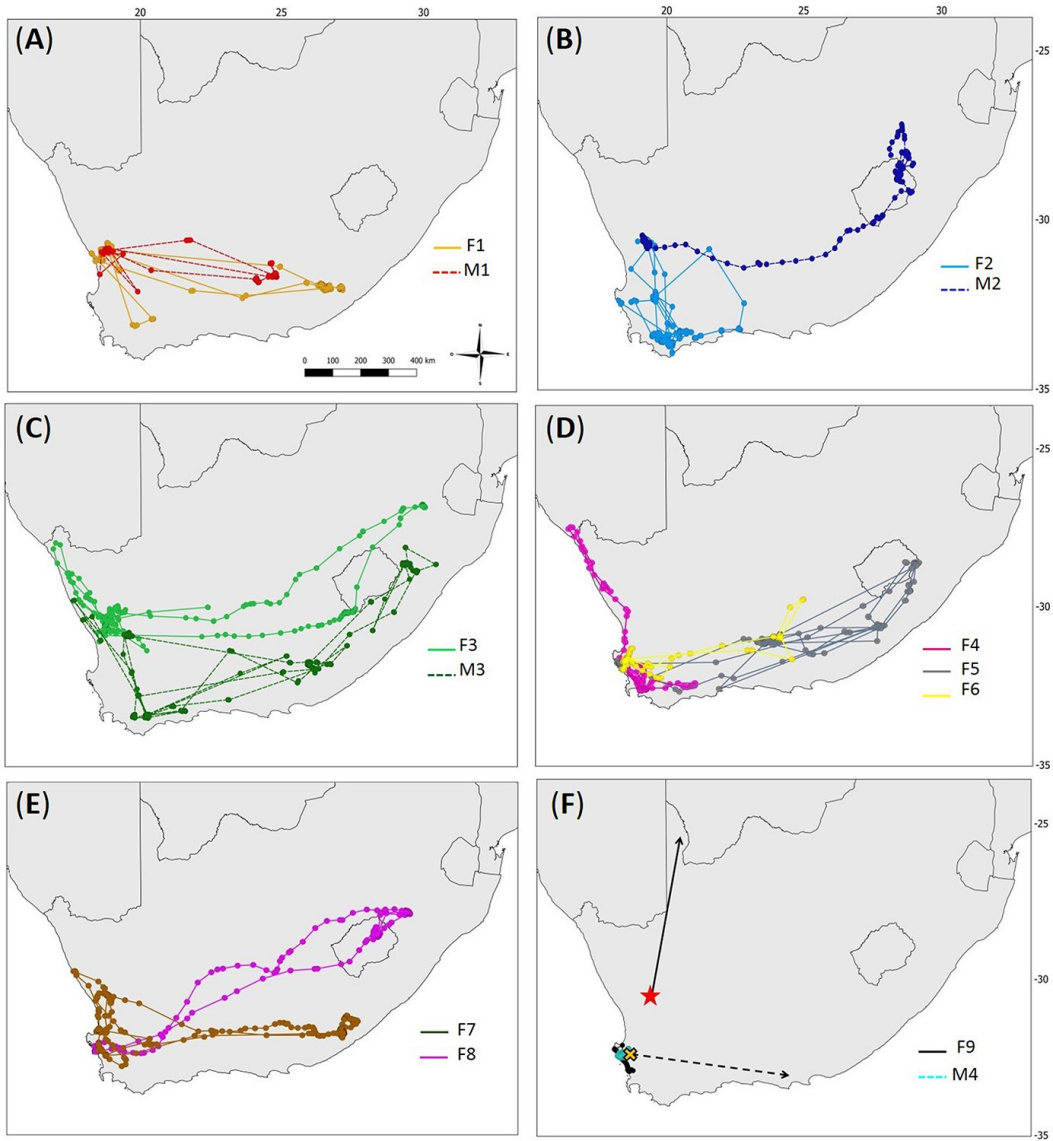
Annual movements of each marked individual Black Harrier in Southern Africa. Thirteen adult Black Harriers were marked with GPS-GSM or PTT tracker devices and followed in south-western South Africa during the 2008–2016 period. Females (F1-F9) are shown with colored solid lines, and males (M1-M4) with colored dashed lines. Plain colored circles represent the satellite locations. Color-codes match those in Fig 1. (**A**) breeding pair F1 and M1; (**B**) breeding pair F2 and M2; (**C**) breeding pair F3 and M3; (**D**) F4, F5, and F6; (**E**) F7 and F8, (**F**) F9 and M4. The two black arrows on panel (**F**) show re-sightings of color-ringed birds during the non-breeding season: breeding adult female marked at nest (red full star) and re-sighted up north a few months later (solid line), and a nestling female marked at nest (orange cross with black contour) and re-sighted at a roost as an adult two years later (dashed line).

Black Harrier annual movements covered the southern half of the land surface of South Africa, including the mountain kingdom of Lesotho (Fig 1A) with marked inter-individual variations in migratory patterns (Fig 2). Most birds (69.2%; 9 birds and 14 post-breeding migrations in total) undertook a long-distance West-East migration. These individuals migrated from south-western South Africa towards the Eastern Cape (i.e. mostly around the Stutterheim area), the south-west region of Kwazulu-Natal (i.e. Mooi River area), the south-west of Mpumalanga, and the north-east of Lesotho (Fig 2, S1 Table, S2 Table). Among them, one female (F6) travelled 1105 km towards the Eastern Cape where she spent a total of 11 days before returning to her former breeding area where she spent the rest of the non-breeding season. Among the medium-distance travelers, two females (F2 and F4; 15% of monitored birds, 3 post-breeding migrations in total) undertook migration to the Overberg, the southern-most part of the Western Cape (Fig 2, S1 Table). Finally, two birds (1 male and 1 female; 15% of monitored birds) behaved as short-migrant/resident (Fig 2, S1 Table): the male M4 stayed in its breeding area (i.e. West Coast National Park, Western Cape) for 287 days, of which 200 were considered as part of its non-breeding season settlement. The second female (F9) moved about 80 km north from her breeding area (Koeberg Private Reserve) to the West Coast National Park where she spent 59 days before returning to Koeberg Private Reserve, where she spent 83 days (Fig 2F). Strikingly, none of the 3 marked pairs followed the same migration routes: pair members did not travel together, nor did they use the same non-breeding areas (Fig 2A, 2B and 2C). Moreover, we obtained two sightings of color-ringed birds during the non-breeding season: 1) a breeding female ringed in November 2013 was sighted about 500–600 km north from her breeding site in the arid Kgalagadi Transfrontier Park in February 2014; 2) a nestling female ringed in the West Coast National Park in October 2014 was re-sighted at a roost two years later 630 km east in the Eastern Cape (Fig 2F).

Overall, we obtained data on 19 post-breeding migrations from 12 birds (excluding the resident male M4). The mean departure date for the post-breeding migrations in the austral summer was January 7^th^ ± 31 days (range: November 24^th^—March 30^th^), with migrants arriving in their first non-breeding area on January 11^th^ ± 31 days (range: December 07^th^—April 4^th^). Successful breeders left significantly later than unsuccessful breeders or non-breeders (χ^2^ = 8.65, d.f. = 1, p = 0.03), with an average difference of 24 days. The mean departure dates of the same breeding individuals during two consecutive years varied by 9–24 days (n = 4 migration events, 2 individuals; S1 Table).

Compared with post-breeding migration events, there were fewer pre-breeding migration events (n = 10 events from 9 birds; S1 Table). The mean departure date for pre-breeding migrations was at the end of the austral winter on August 1^st^ ± 5 days (range: July 22^nd^—August 6^th^), with individuals arriving on the breeding areas on August 9^th^ ± 5 days (range: July 31^st^—August 16^th^).

Distances travelled by migratory Black Harriers did not differ between post- and pre-breeding migrations (respectively, 740 ± 318 km; range: 196–1209 km; and 888 ± 329 km, range: 398–1429 km; Table 1, S1 Table). However, the duration of the winter/spring pre-breeding migrations (6.5 ± 1.9 days, range: 4–9 days) was significantly longer than that of the summer post-breeding migrations (4.6 ± 3.4 days; range: 1–16 days, Table 1, S1 Table). Accordingly, the average daily speed was significantly faster for summer post-breeding migrations than for winter/spring pre-breeding migrations (respectively, 207.8 ± 113.2 km.day^-1^, range: 32–368.3 km.day^-1^, and 143.8 ± 32.2 km.day^-1^, range: 90.9–183.3 km.day^-1^; Table 1, S1 Table). The total number of travel days was not related to the total distance travelled (r = 0.04, p = 0.87, n = 19).

**Table 1:** Summary of the results from the GLMMs testing for the effects of migration events and gender on the duration, distance travelled and daily speed of migration events; as well as testing for the effects of the type of device, sex, number of daily locations, and individual stage on home range sizes.

Independent variables	Explanatory variables	Chi-square	d.f.	P	Estimate
Distance travelled (*n* = 30)	Migration event	2.77	1	0.10	
	Sex	0.56	1	0.45	
Duration* (*n* = 30)					Intercept: 1.53 ± 0.12
	**Migration event**	8.89	1	0.003	0.54 ± 0.18 (pre-breed.**)
	Sex	1.05	1	0.31	
Daily speed (*n* = 30)					Intercept: 228.90 ± 30.44
	**Migration event**	7.65	1	0.01	-78.74 ± 28.48 (pre-breed.**)
	Sex	1.65	1	0.20	
HR 90%* (*n* = 53)					Intercept: 4.54 ± 0.36
	**Device**	8.79	1	0.003	-1.48 ± 0.50 (GPS)
	Sex	0.05	1	0.83	
	Number of daily locations	0.45	1	0.50	
	Individual stage	1.15	2	0.56	
HR 90%*^1^, (*n* = 22)	Sex	0.25	1	0.62	
	Number of daily location	1.12	1	0.29	
	Individual stage	1.73	1	0.42	
HR 90%*^2^, (*n* = 31)	Sex	0.28	1	0.60	
	Number of daily location	0.40	1	0.53	
	Individual stage	0.41	2	0.82	

*Log-transformed

** Pre-breeding (winter/spring) migration

^1^GPS subset

^2^PTT subset

The tested interactions were not included in the table as none were significant. Separate models with data coming from GPS-GSM and PTT tracker devices were also conducted. Individual ID was included as random effects in all models. Significant variables are highlighted in bold, and parameter estimates for those variables are also included. d.f. = degrees of freedom. HR 90% = Home Range 90% kernel. We present the parameter estimates (intercept and estimate of the non-reference term, in brackets, in case of categorical variables) of the final models (those including only significant variables).

### Prospecting movements and breeding dispersal

Great individual variability was apparent in the movements among birds returning from their non-breeding areas to their breeding areas (10 migration events from 9 birds). Some individuals started a pre-breeding prospecting behavior (n = 7 birds, 7 migration events) lasting on average 25.5 ± 6 days. In such cases, individuals’ locations were not clustered around a centroid as birds moved continuously, sometimes for hundreds of kilometers. Some of these first prospected through their former breeding area (n = 5) before eventually settling in the surroundings (n = 2; birds F1 and M1), while others did not stop at their former breeding area but kept prospecting before eventually settling hundreds of kilometers away (n = 3; birds F7, F3, and F4). An extreme case was the female F4, which after a non-breeding season in the Overberg, started pre-breeding prospecting movements, passed through her former breeding area in the Western Cape, travelled about 1135 km north to Namibia, before turning back south towards the Northern Cape (Fig 2C), where the satellite transmitter stopped emitting for unknown reasons. Female F3 exhibited similar behavior, passing through her former breeding area and from there, travelled about 700 km north towards Namibia before turning back to her former breeding area to settle later there (Fig 2C). Other birds (n = 2; birds M3 and F5 for her second monitoring year) also showed a pre-breeding prospecting behavior, but never passed through their former breeding area, to finally settle hundreds of kilometers from it. Finally, some birds did not prospect (n = 3 birds, 3 migration events) and started a breeding-like behavior upon arrival at their former breeding area (i.e. birds F2 and F8 for their second monitoring year, F5 for her third monitoring year).

Only two birds (F2 and F5 during their respective second monitoring year) were confirmed to breed in the year after their initial marking, their nests being respectively located 230 km and 530 km from their previous breeding areas. Female F2 moved from the West Coast National Park (Western Cape) in 2012 to the Nieuwoudtville area (Northern Cape) in 2013, and female F5 moved from the West Coast National Park in 2010 to the Camdeboo Mountains (Eastern Cape) in 2011. Interestingly, the latter area was also used as a non-breeding settlement area by male M1 in 2014.

Only one of three marked pairs returned to the same breeding area in the following year (birds F1 and M1, Fig 2A) although breeding was not confirmed (suspected non-breeders).

Re-sightings of ringed birds indicate that three adult birds bred in the same area where they were ringed as nestlings (e.g. Koeberg Private Reserve, West Coast National Park, *authors unpublished data*).

### Breeding and non-breeding settlement areas

A total of 33 different settlement areas was identified during the non-breeding seasons 2008–2016, with the same individual using up to 3 different areas (1.9 ± 0.8 areas; n = 10 birds using at least 2 settlements) per non-breeding season (Table 2). Individuals stayed on average 77.6 ± 53.5 days in settlement areas (range: 11–206 days), moving within a radius of 11.9 ± 7.6 km (range: 2.4–35.6 km) from the settlement´s centroid. Home range sizes at this stage averaged 163.4 ± 195.1 km^2^ (all birds combined; n = 31). Whereas most individuals exploited the whole area of their settlements equally, four birds (F1, F4, F7, and M3) used more specific sites within their settlement areas and had larger overall home ranges (Table 2, S2 Table). The average distance between two non-breeding settlement areas used successively by the same individual was 212.6 ± 47 km (range: 47–546 km). Those individuals that were monitored for more than one non-breeding season (n = 4, 9 non-breeding seasons) showed a high degree of fidelity to their non-breeding areas among years (Table 2, S2 Table).

**Table 2:** Summary data of the identified breeding and non-breeding settlement areas used by adult Black Harriers.

Name (color)	Device	Number of monitored days	Season	Number of Settlement areas	Total number of locations	Total number of days	Number of daily locations	Average 90% Home Range (km^2^)
F1(orange)	PTT	444	Breeding	2	97	84	1.15	365.37
			Non-breeding	3	250	245	1.02	347.05*
F2 (light blue)	PTT	546	Breeding	2	171	129	1.32	188.79
			Non-breeding	2	459	305	1.50	64.45
F3 (light green)	GPS	418	Breeding	2	1860	208	8.94	52.83
			Non-breeding	3	1015	165	6.15	18.08
F4 (pink)	GPS	233	Breeding	1	482	67	7.19	5.39
			Non-breeding	2	1068	200	5.34	120.91*
F5 (dark grey)	PTT	819	Breeding	3	297	374	0.79	161.93
			Non-breeding	5	274	294	0.93	150.28
F6 (yellow)	GPS	56	Breeding	1	116	10	11.60	18.87
			Non-breeding	2	143	23	6.21	122.96
F7 (brown)	GPS	466	Breeding	2	1685	150	11.23	20.19
			Non-breeding	2	1642	258	6.36	218.89*
F8 (fuchsia)	GPS	306	Breeding	2	827	87	9.51	30.51
			Non-breeding	2	927	197	4.71	90.59
F9 (black)	PTT	198	Breeding	1	27	40	0.68	122.55
			Non-breeding	2	24	142	0.17	48.16
M1 (red)	PTT	395	Breeding	2	156	84	1.86	254.47
			Non-breeding	2	108	95	1.14	185.49
M2 (dark blue)	GPS	132	Breeding	1	1104	63	17.52	110.53
			Non-breeding	1	390	48	8.13	8.02
M3 (dark green)	PTT	447	Breeding	2	178	153		103.08
			Non-breeding	3	172	185	0.93	335.82*
M4 (turquoise)	PTT	287	Breeding	1	93	87	1.07	94.83
			Non-breeding	1	220	200	1.1	336.58

*individuals that used 2–3 locations within a specific settlement area

Thirteen adult Black Harriers were marked with GPS-GSM (n = 6) or PTTs (n = 7) tracker devices and followed in south-western South Africa during the 2008–2016 period. Adult females: F1-F9, adult males: M1-M4. Note that the color-codes in brackets match those of Figs 1 and 2.

Confirmed breeders (n = 13, 15 breeding events) moved within a distance of 16.4 ± 6.8 km (range: 7.1–33.4 km) around their nest. Individuals that were suspected non-breeders in the year after marking (n = 5) moved within an average distance of 18.1 ± 14.4 km (range: 5.5–41.7 km) from their settlement area centroid. Average home ranges at this stage were 92.7 ± 66.6 km^2^ and 147.8 ± 205.4 km^2^ for confirmed breeders (n = 15) and non-breeders (n = 7), respectively.

Home range sizes did not differ significantly among individual stages, between sexes or in relation to the number of daily locations, but they differed according to the type of device (Table 1): birds marked with GPS-GSM trackers had smaller home ranges than those marked with PTT tracker devices (71.9 ± 84.7 km^2^, n = 21 and 187.6 ± 196.5 km^2^, n = 31, respectively, Table 2, S2 Table). No significant interactions were found between type of device and individual stage (χ^2^ = 1.55, d.f. = 2, p = 0.46), the type of device and the number daily locations (χ^2^ = 0.09, d.f. = 1, p = 0.77), or sex and individual stage (χ^2^ = 0.65, d.f. = 2, p = 0.72) (Table 1). Similarly, when looking at data from GPS-GSMs and PTTs separately, the results of GLMMs showed that estimated home range sizes did not vary with the number of daily locations or differ between sexes or among individual stages (Table 1).

### End of signals and mortality cases

Signals ceased at different times of the Black Harriers’ annual cycle (S1 Table) and at different geographical locations. The transmission was lost for 8 of the 13 adults during the non-breeding season, with six birds being located last in the Eastern Cape, Lesotho, or both (F1, F5, F6, F7, M1, and M2), one in the Overberg (F2) and one near the West Coast National Park (M4). The last location of three birds occurred during migration events: two during the summer post-breeding migration (F3 and M3) and one during the winter/spring pre-breeding migration (F4). Only one bird (F8) was lost in the breeding area.

While most of the transmitters (n = 10) stopped emitting for unknown reasons, we confirmed the death of three birds by finding their carcasses in the field and collecting the devices: female F6 was killed within her non-breeding settlement area after a collision with a power line (i.e. an X-ray showed a fractured spine), female F8 was killed by an apparent poisoning (possibly by rodenticides) at her former breeding area, a few days after she returned from her pre-breeding migration; and male M4 was found dead near power lines within his non-breeding season settlement area, although the actual cause of death remained unknown. Additionally, the carcass of a marked adult male (ringed as nestling in 2013 in the same area), was found on the side of a national road in November 2018: this bird showed all the evidences to being hit by a car (i.e. typical fracture of the neck).

## Discussion

### Migratory movements

Black Harrier’s migration routes covered the entire southern land area of South Africa and Lesotho, but, exhibited great individual variability in migratory patterns. Long-distance migrants performed an unusual West-East migration, with individuals travelling eastwards from their breeding areas in south-western South Africa to the Eastern Cape, Kwazulu-Natal, Mpumalanga, and through Lesotho during the austral summer (Fig 1, S2 Table). Such East-West migration patterns have been described in the North American Prairie Falcon (*Falco mexicanus*) [16], but remains unusual for migratory birds of prey, which typically migrate North-South [14]. As migratory movements essentially occur in response to seasonal changes in food resources [2], it is reasonable to suggest that Black Harriers initiate migration to cope with declines in food availability (i.e. accessibility and/or abundance of prey) in their breeding areas at the end of the breeding season. Breeding Black Harriers are small-mammal specialists, essentially feeding on Striped Mouse (*Rhabdomys pumilio*), although they also feed on birds and lizards as alternative prey [36]. The abundance of small mammals is known to fluctuate strongly with rainfall regimes (i.e. higher abundance occurs with greater rainfall) and with temperature regimes [53]. Ultimately this may induce reduced movements of prey during the hottest time of the day and greater activity at dusk and dawn [38, 54–56]. We recently showed that Black Harriers breeding inland in the Karoo biome were forced to shift, as their breeding season progressed, from their primary prey (i.e. small mammals) to less favorable alternative prey (i.e. birds and lizards), probably due to a temperature-mediated decrease of Striped Mice availability at that time [36]. As the climate in south-western South Africa (i.e. Black Harriers’ breeding range) is Mediterranean-like, a lack of rainfall and high temperatures in summer (i.e. from November to March) [57] could further alter the activity and abundance of Striped Mice making these even less available for Black Harriers later in the season. In contrast, the eastern part of South Africa (i.e. Eastern Cape, Kwazulu Natal) and Lesotho receives rainfall from November to February [57], coinciding with the migratory movements of Black Harriers there. Overall, grassland-type and mixed crop-natural vegetation within the latter regions are known to maintain high abundances of small mammal species of the *Rhabdomys* taxa during summer months [58]. Additionally, the mountainous region of Lesotho is known to sustain abundant populations of Ice Rats, e.g. *Otomys sloggetti robertsi*, another important prey for Black Harriers (R. E. Simmons, *unpublished data*), which increase to maximum population size as Black Harriers arrive in February-May. We thus suggest that the Black Harrier acts like other small-mammal specialists [59] and migrates (East) to exploit the seasonal abundance and accessibility of its primary prey, and/or more profitable prey such as Ice Rats in Lesotho [60].

Our results show that adult Black Harriers travel almost twice as fast during summer post-breeding migrations as during winter/spring pre-breeding migrations, and not the other way around as reported in other species [13, 14, 61–63]. This pattern might be associated with their marked pre-breeding prospecting behavior and their large dispersal distances among consecutive breeding events in an apparent attempt to find the best breeding areas. Given that breeding areas are widespread (including some of those used during the non-breeding season, see Fig 1B) returning adults may take time looking for the most suitable areas for breeding (i.e. habitats with good vegetation cover to reduce predation risk at nests, and high prey availability and abundance for good food resource; see [34–36]). These may be located hundreds of kilometers away from non-breeding or previous breeding areas. Similar behavior has been found in another small mammal harrier specialist, the Pallid Harrier [64], and may indicate a tendency for nomadism associated with rodent prey specialization. Additionally, migrants returning to their breeding areas may be constrained by weather conditions, such as wind drift [65]. Indeed, during their pre-breeding migrations, Black Harriers returning to the breeding areas may face dominant westerly winds, forcing individuals to take more breaks e*n route*, whereas during post-breeding migrations, individuals would benefit from these prevailing winds and travel faster. This has been described in other migratory species [14, 66, 67], but would need to be tested in the case of Black Harriers.

### Breeding dispersal and fidelity rates

Although based on a very small sample size, our observations from re-sightings of ringed birds suggest that Black Harriers show some degree of fidelity to breeding areas and natal philopatry. However, results also show that large breeding dispersal may occur, representing an important aspect of its life-history to account for when devising management or conservation programs. This, linked with the pre-breeding prospecting behavior recorded for this small-mammal specialist, agrees with what has been observed in other irruptive species such as the Snowy Owl (*Bubo scandiacus*) [68] or the Pallid Harrier [69].

All tagged birds returned to breeding areas (n = 11) and showed a breeding-like behavior in the year following marking, but only two individuals were confirmed breeding. This may have several explanations: (i) individuals initiated breeding but failed at an early stage before breeding could be confirmed; (ii) environmental conditions were not suitable for breeding, e.g. low rainfall regime leading to poor food resources [30], so adults chose not to invest in reproduction [70]; (iii) satellite tagging may have had a negative carry-over effect influencing subsequent ability to breed successfully. We consider the latter unlikely, as this did not occur in other harriers of similar size travelling longer distances and tagged with similar devices [38, 71]; (iv) Black Harriers may take a “sabbatical year” in reproduction if the physiological effort of previous breeding was too high. Such a breeding strategy is found in some long-lived birds such as Eagles [72], Petrels or Albatrosses [73], but further investigation is required to confirm if this regularly occurs in Black Harriers. Confirming individual breeding rate across years will be critical as this may have important implications for population dynamics and sustainability in the long term [1]. Our results also suggest caution is required in assuming that an adult bird is breeding when the latter is based only on spatial movement analyses without direct observations to confirm this.

### Settlement areas and home ranges

Black Harriers showed a high degree of fidelity to non-breeding settlement areas, with adults returning to the same area they settled in the year before (Table 2, S2 Table). Some birds settled in a unique area, whereas others settled in up to three different areas used consecutively within the same non-breeding season (Table 2, S2 Table). This pattern has been described in a few raptors, including Prairie Falcons [16], Montagu’s Harriers [74], and Snowy Owls [75] and appears to reflect temporal variation in local prey abundance during the non-breeding season. It would be interesting in the future to assess diet and prey abundance in these settlement areas used by Black Harriers.

Home ranges during the breeding season (for confirmed breeders or suspected non-breeders) and during the non-breeding seasons were of similar sizes. Typically, breeding individuals show smaller home ranges than non-breeders or birds outside the breeding season, because the former have to defend a fixed territory whereas the latter are less “attached” to a fixed location and usually cover a larger area [76, 77]. Such differences have however, not been found in other species like Montagu´s Harriers, where the size of home ranges is more likely to vary in relation to habitat types and food resources [74]. For instance, larger home ranges late in the wintering season (associated with lower locust availability) have been found in Montagu´s Harriers [78]. In this context, the similar home range sizes between breeding and non-breeding seasons for Black Harriers may suggest that food availability in the breeding and non-breeding areas is similar when the harriers are occupying them. This is expected since the breeding (austral winter/spring) and non-breeding (austral summer/fall) locations both benefit from winter then summer rains, respectively.

Nevertheless, it is important to note that the estimated home range sizes varied significantly with the type of device used: home ranges estimated with PTTs were greater than those from GPS-GSMs. These differences may be explained by the greater location accuracy of GPS devices (< 5 m of accuracy) compared to PTTs (highest accuracy 150 m), suggesting the latter is liable to overestimation. Additional work is needed in this area.

### Conservation implications and future research

Information about movements of Black Harriers throughout the annual cycle should aid conservation management planning for the species, in particular as the species crosses both regional and international borders. For instance, knowledge of the areas used by Black Harriers outside the breeding season can help guide conservation management by protecting areas critical at that time. In this context, it will be important to identify which habitat types Black Harriers use in non-breeding settlement areas, and which prey types this specialist predator relies on during the non-breeding season. Additional information on movements throughout the annual cycle and on home range sizes will also be helpful to map potential risk areas for the species. This could also help to inform the placement of wind turbines in relation to known breeding locations, which are known to cause mortalities in Black Harriers (R.E. Simmons and M. Martins, *unpublished data*) and other threatened raptors in Southern Africa in recent years [79]. Additionally, this could help identifying risk areas where contamination by pesticides (i.e. DDTs) and other contaminants (i.e. PCBs) may be acquired, which are known to induce sub-lethal effects in this species [24, 80].

Moreover, obtaining information about the capacity for long-distance movements between breeding seasons may also help interpret observations in citizen-based programs like SABAP to better understand breeding population sizes and trends. The observation that not all individuals breed every year has implications for understanding the impact of breeding conditions (e.g. rainfall and temperature) on population sustainability and deserves further attention. Finally, the observation that most (84.6% of tagged Black Harriers) putative death (i.e. loss of signal) or confirmed death occurred either during the non-breeding season or during a migration event, parallels findings in other raptors [4], and justifies even more the urgent need to obtain further information on threats throughout the entire annual-cycle, with the ultimate goal of ensuring a sustainable conservation of this endangered species. This may *de facto* serve as a critical first step in the overall conservation of intra-African migrant bird species.

## Supporting information

S1 Table Summary of annual movement data obtained from the 13 adult Black Harriers marked with GPS-GSM or PTT tracker devices and followed in south-western South Africa during the 2008–2016 period. (DOCX)

S2 Table More detailed summary data for the identified breeding and non-breeding settlement areas used by the 13 adult Black Harriers marked with GPS-GSM or PTT tracker devices followed in south-western South Africa during the 2008–2016 period. (DOCX)

## References

[1] Newton I. Population Ecology Of Raptors. Poyser T, Poyser AD edition, Berkhamsted, UK. 1979.

[2] Newton I. The Migration Ecology Of Birds. 1st edition, Academic Press, London, UK 2007.

[3] Rodriguez-Riuz J, Mougeot F, Parejo D, de la Puente J, Bermejo A, Avilés JM. Important areas for the conservation of the European Roller during the non-breeding season in southern Africa. Bird Conservation International. 2018;*in press*.

[4] Klaassen RHG, Hake M, Strandberg R, Koks BJ, Trierweiler C, Exo KM, et al When and where does mortality occur in migratory birds? Direct evidence from long-term satellite tracking of raptors. Journal of Animal Ecology. 2014;83: 176–184. 10.1111/1365-2656.12135 24102110

[5] Norris DR, Marra PP, Kyser TK, Ratcliffe KM. Tropical winter habitat selection limits reproductive success on the temperate breeding grounds in a migratory bird. Proceedings of the Royal Society-series B. 2003;271: 59–64. 10.1098/rspb.2003.2569 PMC1691559 15002772

[6] Gunnarsson TG, Gill JA, Newton J, Potts PM, Sutherland WJ. Seasonal matching of habitat quality and fitness in a migratory bird. Proceedings of the Royal Society B. 2005;272: 2319–2323. 10.1098/rspb.2005.3214 16191646 PMC1560186

[7] Peach WJ, Baillie SR, Underhill L. Survival of British sedge warblers (*Acrocephalus schoenobaenus*) in relation to West African rainfall. Ibis. 1991;133: 300–305.

[8] Dolman PM, Sutherland WJ. The response of bird populations to habitat loss. Ibis. 1994;137:38–48.

[9] Marra PP, Hobson KA, Holmes RT. Linking winter and summer events in a migratory bird by using stable-carbon isotopes. Science. 1998;282:1884–1886. 9836637 10.1126/science.282.5395.1884

[10] Webster MS, Marra PP, Haig SM, Bensch S, Holmes RT. Links between worlds: unraveling migratory connectivity. Trends of Ecology and Evolution. 2002;17: 76–83.

[11] Szép T, MØller AP, Nuttal SPR, Szabó AD, Pap PL. Searching for potential wintering and migration areas of a Danish Barn Swallow population in South Africa by correlating NDVI with survival estimates. Journal of Ornithology. 2006;147: 245–253.

[12] Robinson WD, Browlin MS, Bisson I, Shannon-Barnes S, Thorup K, Diehl RH, et al Integrating concepts and technologies to advance the study of bird migration. Frontiers in Ecology and Environment. 2010;8: 354–361.

[13] Terraube J, Mougeot F, Cornulier T, Verma A, Gavrilov A, Arroyo B. Broad wintering range and intercontinental migratory divide within a core population of the near-threatened Pallid Harrier. Biodiversity and Distribution. 2012;18: 401–409.

[14] Bildstein KL. Migrating Raptors Of The World: Their Ecology And Conservation. Cornell University Press, Ithaca, New York; 2006.

[15] Monti F, Gremillet D, Sforzi A, Sammuri G, Dominici JM, Triay Bagur R, Munoz Navarro A, Fusani L, Duriez O. Migration and wintering strategies in the vulnerable Mediterranean osprey populations. 2018;4: 554–567

[16] Steenhof K, Fuller MR, Kochert MN, Bates KK. Long-range movements and breeding dispersal of Prairie Falcons from Southwest Idaho. The Condor. 2005;107: 481–496.

[17] Dodge S, Bohrer G, Bildstein K, Davidson SC, Weinzierl R, Bechard MJ et al Environmental drivers of variability in the movement ecology of Turkey Vultures (*Cathartes aura*) in North and South America. Philosophical Transactions of the Royal Society B. 2014;369: 20130195. 10.1098/rstb.2013.0195 PMC3983930 24733950

[18] Gschweng M, Kalko EKV, Querner U, Fiedler W, Berthold P. All across Africa: highly individual migration routes of Eleonora’s Falcon. Proceedings of the Royal Society B. 2008;275: 2887–2896. 10.1098/rspb.2008.0575 18765348 PMC2605830

[19] Meyburg B-U, Mendelsohn JM, Ellis DH, Meyburg C, Kemp AC. Year-round movements of a Wahlberg’s Eagle (*Aquila wahlbergi*) tracked by satellite. Ostrich. 1995;66: 135–140.

[20] Paijmans DM, Catto A, Oschaldeus HD. SAFRING longevity and movement records for southern African vultures (subfamilies *Aegypiinae* and *Gypaetinae*). Ostrich. 2017;88: 163–166.

[21] Ogada D, Shaw P, Beyers RL, Buij R, Murn C, Thiollay JM, Beale CM, Holdo RM, Pomeroy D, Baker N, Krüger SC, Botha A, Virani MZ, Monadjem A, Sinclair ARE. Another Continental Vulture Crisis: Africa’s Vultures Collapsing toward Extinction. Conservation Letters. 2016;9: 89–97.

[22] Thiollay JM. The decline of raptors in West Africa: long-term assessment and the role of protected areas. Ibis. 2006;148: 240–54.

[23] Tarboton WR, Allan DG. The status and conservation of birds of prey in the Transvaal. Pretoria, Transvaal Museum. 1984.

[24] Garcia-Heras M-S, Arroyo B, Simmons RE, Camarero PR, Mateo R, Mougeot F. Blood concentrations of PCBs and DDTs in an avian predator endemic to southern Africa: Associations with habitat, electrical transformers and diet. Environmental Pollution. 2018;232:440–449. 10.1016/j.envpol.2017.09.059 28986081

[25] Garbett R, Maude G, Hancock P, Kenny D, Reading R, Amar A. Association between hunting and elevated blood lead levels in the critically endangered African white-backed vulture (*Gyps africanus*). Science of the Total Environment. 2018;630:1654–1665. 10.1016/j.scitotenv.2018.02.220 29550066

[26] Simmons RE, Brown CJ, Kemper J. Birds to watch in Namibia: red, rare and endemic species. Ministry of Environment & Tourism, Windhoek, Namibia. 2015.

[27] Taylor M. Black Harrier (*Circus maurus*) In: Taylor M, Peacock F, Wanless R edition. The 2015 Eskom Red data book of birds of South Africa, Lesotho and Swaziland. Birdlife South Africa Johannesburg; 2015 pp. 125–127.

[28] Curtis O, Simmons RE, Jenkins AR. Black Harrier (*Circus maurus*) of the Fynbos Biome, South Africa: a threatened specialist or an adaptable survivor? Bird Conservation International. 2004;14: 233–245.

[29] Curtis O. Responses of raptors to habitat fragmentation: from individual responses to population susceptibility. MSc Thesis, University of Cape Town, South Africa. 2005.

[30] García-Heras M-S. Environmental factors influencing the breeding and health of a predator endemic to southern Africa: the Endangered Black Harrier (*Circus maurus*). PhD Thesis, FitzPatrick Institute of African Ornithology, University of Cape Town, South Africa; 2017.

[31] Underhill LG, Brooks M. Pentad-scale distribution maps for bird atlas data. Biodiversity Observations. 2016a;7.52: 1–8.

[32] Underhill LG, Brooks M. Displaying changes in bird distributions between SABAP1 and SABAP2. Biodiversity Observations. 2016b;7.62: 1–13.

[33] Fuchs J, Simmons RE, Mindell DP, Bowie RCK, Oatley G. Lack of mtDNA genetic diversity in the Black Harrier (*Circus maurus*), a southern African endemic. Ibis. 2014;156: 227–230.

[34] García-Heras M-S, Arroyo B, Mougeot F, Amar A, Simmons RE. Does timing of breeding matter less where the grass is greener? Seasonal declines in breeding performance differ between regions in an endangered endemic raptor. Nature Conservation. 2016;15:23–46.

[35] García-Heras M-S, Mougeot F, Arroyo B, Avery G, Avery MD, Simmons RE. Is the Black Harrier (*Circus maurus*) a specialist predator? Assessing the diet of a threatened raptor species endemic to Southern Africa. Ostrich: Journal of African Ornithology. 2017a;2017: 1–8.

[36] García-Heras M-S, Mougeot F, Simmons RE, Arroyo B. Regional and temporal variations in diet and provisioning rates suggest weather limits prey availability for an endangered avian predator. Ibis. 2017b;159: 567–579.

[37] García-Heras M-S. Environmental factors influencing the breeding and health of a predator endemic to southern Africa: the endangered Black Harrier Circus Maurus, FitzPatrick Institute of African Ornithology, University of Cape Town, South Africa. PhD thesis. 2017.

[38] Simmons RE. Harriers of The World: Their Behaviour And Ecology. Oxford University Press. 2000.

[39] Steenhof K, Bates KK, Fuller MR, Kochert MN, McKinley JO, Lukacs PM. Effects of radiomarking on Prairie Falcons: attachments failures provide insights about survival. Wildlife Society Bulletin. 2006;34: 116–126.

[40] Limiñana R, Soutullo A, Urios V. Autumn migration of Montagu’s harriers (*Circus pygargus*) tracked by satellite telemetry. Journal of Ornithology. 2007;148: 517–523.

[41] Meyburg B-U, Fuller MR. Satellite Tracking In: Bird DM, Bildstein KL, editors. Raptor Research Management Techniques. Hancock House Publishers, Surrey, Canada 2007 Pp. 242–248.

[42] Hays GC, Arkesson S, Godley BJ, Luschi P, Santidrian P. The implication of location accuracy for the interpretation of satellite-tracking data. Animal behaviour. 2001;61: 1035–1040.

[43] Arroyo B, Leckie F, Amar A, Mccluskie A, Redpath S. Ranging behaviour of Hen Harriers breeding in Special Protection areas in Scotland. Bird Study. 2014;61: 48–55.

[44] QGIS Development Team. QGIS Geographic Information System. Open Source Geospatial Foundation. 2015.

[45] Worton BJ. Kernel methods for estimating the utilization distribution in home-range studies. Ecology. 1989;70: 164–168.

[46] Worton BJ. Using Mote Carlo simulation to evaluate kernel-based home-range estimators. Journal of wildlife Management. 1995;59: 794–800.

[47] Kenward RE, Clarke RT, Hodder KH, Walls SS. Density and linkage estimators of home-range: nearest-neighbor clustering defines multi-nuclear cores. Ecology. 2001;82: 1905–1920.

[48] Calenge C. The package “adehabitat” for the R software: a tool for the analysis of space and habitat use by animals. Ecological Modelling. 2006;197: 516–9.

[49] Calenge C. Home range Estimation in R: the “adehabitatHR” package; 2011.

[50] Bivand R, Keitt R, Rowlingson B. rdal: bindings for the geospatial data abstraction library. R package version 0.9–1; 2014.

[51] The R foundation for statistical computing,”Wooden Christmas-Tree”, copyright Platform: x86_64-w64-mingw32/x64 (64-bit). 2015.

[52] Bates D, Maechler M, Bolker B, Walker S, Christensen RHB, Singmann H, et al Lme4: Linear mixed–effects models using S4 classes. R package ver. 0.999999–0; 2012.

[53] Rymer TL, Pillay N, Schradin C. Extinction or survival? Behavioral flexibility in response to environmental change in the African Striped mouse *Rhabdomys*. Sustainability. 2013;5: 163–186.

[54] Perrin MR. Notes on the activity of 12 species of Southern African rodents and a new design of activity monitor. South African Journal of Zoology. 1981;16: 248–258.

[55] Schradin C, Pillay N. The Striped Mouse (*Rhabdomys pumilio*) from the succulent Karoo, South Africa: a territorial group-living solitary forager with communal breeding and helpers at the nest. Journal of Comparative Psychology. 2004;118: 37–47. 10.1037/0735-7036.118.1.37 15008671

[56] Nater C, Canale CI, Van Benthem KJ, Yuen CH, Schoepf I, Pillay N, et al Interactive effects of exogenous and endogenous factors on demographic rates of an African rodent. Oikos. 2016;125: 1838–1848.

[57] Mucina L, Rutherford MC. The Vegetation Of South Africa, Lesotho And Swaziland. South African National Biodiversity Institute, Pretoria, South Africa 2006.

[58] Meynard CN, Pillay N, Perrigault M, Caminade P, Ganem G. Evidence of environmental niche differentiation in the Striped Mouse *Rhabdomys* sp.: inference from its current distribution in southern Africa. Ecology and Evolution. 2012;2: 1008–1023. 10.1002/ece3.219 22837845 PMC3399166

[59] Newton I. Advances in the study of irruptive migration. Ardea. 2006;94: 433–460.

[60] Mokotjomela T, Schwaibold U, Pillay N. Population Surveys of the Ice Rat (*Otomys Sloggetti Robertsi*) in the Lesotho Drakensberg. African Zoology. 2010;45: 225–232.

[61] Berthold P. Bird Migration: A General Survey. 2snd ed Oxford Ornithology Series no.12, Oxford University Press New York; 2001.

[62] McGrady MJ, Maechtle TL, Vargas JJ, Seegar WS, Pena PP. Migration and ranging of Peregrine Falcons wintering on the Gulf of Mexico coast, Tamaulipas, Mexico. The Condor. 2002;104: 39–48.

[63] Alerstam T, Hake M, Kjelle´n N. Temporal and spatial patterns of repeated migratory journeys by Ospreys. Animal Behaviour. 2006;71: 555–566.

[64] Terraube J. Integrating foraging strategies, spatial movement patterns and reproductive success: implication for the conservation of sympatric avian predators. PhD Thesis, Instituto de Investigación en Recursos Cinegéticos (CSIC-UCLM-JCCM), Ciudad Real, Spain. 2011.

[65] Kruger AC, Goliger AM, Retied JV, Sekele S. Strong wind climatic zones in South Africa Wind and Structures. 2010;13: 000–000.

[66] Klaassen RHG, Hake M, Strandberg R, Alerstam T. Geographical and temporal flexibility in the response to crosswinds by migrating raptors. Proceedings of the Royal Society B. 2011;278: 1339–1346. 10.1098/rspb.2010.2106 20980299 PMC3061148

[67] Mellone U, Klaassen RHG, García-Ripollés C, Limiñana R, López-López P, Pavón D, et al Interspecific comparison of the performance of soaring migrants in relation to morphology, meteorological conditions and migration strategies. PLoS ONE. 2012;7(7): e39833 10.1371/journal.pone.0039833 22768314 PMC3388085

[68] Therrien JF, Gauthier G, Pinaud D, Bêty J. Irruptive movements and breeding dispersal of snowy owls: a specialized predator exploiting a pulsed resource. Journal of Avian Biology. 2014;45: 536–544.

[69] Terraube J, Arroyo B, Bragin A, Bragin E, Mougeot F. Ecological factors influencing the breeding distribution and success of a nomadic, specialist predator. Biodiversity & Conservation. 2012;21: 1835–1852.

[70] Arroyo B, Bretagnolle V, Leroux ALA. Interactive effects of food and age on breeding in the Montagu’s Harrier *Circus pygargus*. Ibis. 2007;149: 806–813.

[71] Trierweiler C. Travels to feed and food to breed: the annual cycle of a migratory raptor, Montagu’s Harrier, in a modern world. PhD Thesis, University of Groningen, Netherlands. 2010.

[72] Simmons RE. Why don't all siblicidal eagles lay insurance eggs? The egg quality hypothesis. Behavioral Ecology. 1997;8: 544–550.

[73] del Hoyo J, Elliott A, Sargatal J. Handbook Of The Birds Of The World–Volume 1, Lynx Editions, Barcelona, Spain; 1992.

[74] Trierweiler C, Mullié WC, Drent RH, Exo K-M, Komdeur J, Bairlein F, et al A Paleartic migratory raptor species tracks shifting prey availability within its wintering range in the Sahel. Journal of Animal Ecology. 2013;82: 107–120. 10.1111/j.1365-2656.2012.02036.x 23137184

[75] Robillard A, Gauthier G, Therrien JF, Bety J. Wintering space use and site fidelity in a nomadic species, the Snowy Owl. Journal of Avian Biology. 2018;e01707.

[76] Peery MZ. Factors affecting interspecies variation in home-range size of raptors. The Auk. 2000;117: 511–517.

[77] Bosch R, Real R., Tinto AL, Zozaya EL, Castell C. Home ranges and patterns of spatial use in territorial Bonelli´s Eagles (*Aquila fasciata*). Ibis. 2010;152: 105–117.

[78] Schlaich AE, Klaassen RHG, Bouten W, Bretagnolle V, Koks BJ, Villers A et al How individual Montagu’s Harriers cope with Moreau’s paradox during the Sahelian winter. Journal of Animal Ecology. 2016;85: 1491–1501. 10.1111/1365-2656.12583 27642032

[79] Reid T, Kruger S, Whitfield DP, Amar A. Using spatial analyses of Bearbed Vultures movements in southern Africa to inform wind turbine placement. Journal of Applied Ecology. 2015;52: 881–892.

[80] García-Heras M-S, Arroyo B, Simmons RE, Camarero PR, Mateo R, García JT, et al Pollutants and diet influence carotenoid levels and integument coloration in nestlings of an endangered raptor. Science of the Total Environment 2017c;603–604: 299–307. 10.1016/j.scitotenv.2017.06.048 28628821

