# Supplementary material for: Migratory patterns and settlement areas revealed by remote sensing in an endangered intra-African migrant, the Black Harrier (Circus maurus)
Source: PLoS One. 2019 Jan 17;14(1):e0210756. doi: 10.1371/journal.pone.0210756 (PMC6336274; doi:10.1371/journal.pone.0210756)
Supplement: S1 Table — (DOCX) [file pone.0210756.s001.docx]

**Supporting Information**

Migratory patterns and settlement areas revealed by remote sensing in an endangered intra-African migrant, the Black Harrier *(Circus maurus);*

Marie-Sophie Garcia-Heras*^1,2*^*, Beatriz Arroyo^3^*^#^*, François Mougeot*^3#^*, Keith Bildstein*^4^*, Jean-François Therrien*^4^* & Robert E. Simmons^2#^

**S1 Table: Summary of annual movement data obtained from the 13 adult Black Harriers marked with GPS-GSM or PTT tracker devices and followed in south-western South Africa during the 2008-2016 period.**

| Name  (color on Figures 1&2) | Marked mate, year | Device | Start of transmission - End of signal  (total followed days) | Type of annual movement  ()^δ^ | Start of migration event | End of migration Event | Migration duration (days) | Total distance (km) | Average daily speed (km.day^-1^) |
| --- | --- | --- | --- | --- | --- | --- | --- | --- | --- |
| F1  (orange) | M1, 2013 | PTT | 2013/11/21 - 2015/01/28  (444) | Post-breeding migration1 (LD) | 2013/12/25 - 2013/12/27 [26]* | 2013/12/30 - 2014/01/01 [31]* | 5 | 822 | 164.4 |
|  |  |  |  | Pre-breeding migration1 (LD) | 2014/07/30-2014/08/03 [1]* | 2014/08/08 - 2014/08/11 [10]** | 9 | 985 | 109.4 |
|  |  |  |  | Post-breeding migration2 (LD) | 2014/12/03-2014/12/05 [4]* | 2014/12/05-2014/12/08 [7]** | 3 | 826 | 275.3 |
| F2  (light blue) | M2, 2013 | PTT | 2012/12/14 - 2014/06/12  (546) | Post-breeding migration1  (MD) | 2012/12/21 | 2012/12/22 | 1 | 206 | 206 |
|  |  |  |  | Pre-breeding migration1  (MD) | 2013/07/21-2013/07/23 [22]* | 2013/07/30- 2013/08/11 [6]** | 15 | 398 | 26.5 |
|  |  |  |  | Post-breeding migration2 (MD) | 2013/12/11 -2013/12/12 [12]** | 2013/12/14-2013/12/15 [15]** | 3 | 384 | 128 |
| F3  (light green) | M3, 2013 | GPS | 2013/11/07 - 2015/01/01  (418) | Post-breeding migration1 (LD) | 2014/03/07 | 2014/03/09 - 2014/03/10 [10]** | 3 | 786 | 262 |
|  |  |  |  | Pre-breeding migration1 (LD) | 2014/08/01 | 2014/08/07 - 2014/08/08 [08]** | 7 | 1283 | 183.3 |
|  |  |  |  | Post-breeding migration2 (LD) | 2014/12/31 | 2015/01/01 – Ø | 1 | 366 | 366 |
| F4  (pink) | _ | GPS | 2014/10/28 - 2015/08/20  (233) | Post-breeding migration1 (LD) | 2015/03/30 | 2015/04/03 | 4 | 196 | 49 |
|  |  |  |  | Pre-breeding migration1 (LD) | 2015/08/05 | 2015/08/16 | 11 | 1135 | 103.2 |
| F5  (dark grey) | _ | PTT | 2010/11/17 - 2013/02/11  (819) | Post-breeding migration1 (LD) | 2011/02/03 -2011/02/05 [4]* | 2011/02/08 - 2011/02/13 [11]** | 7 | 1189 | 169.9 |
|  |  |  |  | Pre-breeding migration1 (LD) | 2011/08/05 - 2011/08/06 [6]** | 2011/08/11 - 2011/08/15 [13]* | 7 | 636 | 90.9 |
|  |  |  |  | Post-breeding migration2 (LD) | 2012/01/10 - 2012/01/11 [11]** | 2012/01/16 | 5 | 789 | 157.8 |
|  |  |  |  | Pre-breeding migration2 (LD) | 2012/07/22 - 2012/07/30 [26]* | 2012/07/30-2012/08/01 [31]* | 5 | 887 | 177.4 |
|  |  |  |  | Post-breeding migration3 (LD) | 2012/12/30 | 2013/01/04 - 2013/01/05 [5]** | 6 | 617 | 102.8 |
| F6^ǂ^  (yellow) | _ | GPS | 2015/11/14 - 2016/01/08  (56) | Post-breeding migration1 (LD)^Ʒ^ | 2015/12/08 | 2015/12/11 | 3 | 1105 | 368.3 |
| F7  (brown) | _ | GPS | 2013/11/06 - 2015/02/14  (466) | Post-breeding migration1 (LD) | 2014/01/09 | 2014/01/12 | 3 | 913 | 304.33 |
|  |  |  |  | Pre-breeding migration1 (LD) | 2014/08/06 | 2014/08/10 - 2014/08/11 [11] | 5 | 860 | 172 |
|  |  |  |  | Post-breeding migration2 (LD) | 2014/12/20 - 2014/12/21 [21]** | 2014/12/22 - 2014/12/24 [23]* | 2 | 860 | 430 |
| F8^ǂ^  (fuchsia) | _ | GPS | 2014/10/23 - 2015/08/25  (306) | Post-breeding migration1 (LD) | 2015/01/03 - 2015/01/04 [04]** | 2015/01/09 | 5 | 1209 | 241.8 |
|  |  |  |  | Pre-breeding migration1 (LD) | 2015/07/31 -2015/08/01 [01]** | 2015/08/10 | 9 | 1429 | 158.8 |
| F9^ǂ^  (black) | _ | PTT | 2008/12/03 - 2009/06/19  (198) | Post-breeding migration1 (SD/R) | 2009/01/10-2009/01/14 [12]* | 2009/01/19-2009/01/23 [21]** | 9 | 288^Ʊ^ | 32 |
| M1  (red) | F1, 2013 | PTT | 2013/11/21 - 2014/12/10  (395) | Post-breeding migration1 (LD) | 2014/01/23 - 2014/01/25 [24]* | 2014/01/26 - 2014/01/28 [27]* | 3 | 625 | 208.3 |
|  |  |  |  | Pre-breeding migration1 (LD) | 2014/07/24 - 2014/07/29 [27]** | 2014/07/29 - 2014/08/02 [31]* | 4 | 546 | 136.5 |
|  |  |  |  | Post-breeding migration2 (LD) | 2014/11/18 - 2014/11/30 [24]* | 2014/12/10 | 16 | 597 | 37.3 |
| M2  (dark blue) | F2, 2013 | GPS | 2013/11/06 - 2014/03/20  (132) | Post-breeding migration1 (LD) | 2014/01/14 - 2014/01/15 [15]** | 2014/01/19 - 2014/01/20 [20]** | 5 | 1185 | 237 |
| M3  (dark green) | F3, 2013 | PTT | 2013/11/07 - 2015/01/29 (447) | Post-breeding migration1 (LD) | 2013/12/23-2013/12/25 [24]* | 2013/12/26-2013/12/28 [27]* | 3 | 653 | 217.7 |
|  |  |  |  | Pre-breeding migration1 (LD) | 2014/08/04-2014/08/06 [05]* | 2014/08/11 | 6 | 722 | 120.3 |
| M4  (turquoise) | _ | PTT | 2008/10/12-2009/07/27  (287) | No migration, (SD/R) | _ | _ | _ | _ | _ |

^δ^LD: Long-distance, MD: Medium-distance, SD/R: Short-distance/resident

[-]* mean of the range of dates

[-]**rounded up mean of the range of dates, as mean was not an exact number

ǂ Found dead body

Ø device stops emitting *en route*

^Ʒ^ conducted a reversed migration back to her breeding areas on 2015/12/22 after spending 11 days in the Eastern Cape Province

^Ʊ^ distance including prospections as trip from breeding to non-breeding area was not direct (~ 80 km is the actual distance between both settlements)

Adult females: F1-F9, adult males: M1-M4. All dates are given in the format Year/Month/Day.
