# Supplementary material for: Migratory patterns and settlement areas revealed by remote sensing in an endangered intra-African migrant, the Black Harrier (Circus maurus)
Source: PLoS One. 2019 Jan 17;14(1):e0210756. doi: 10.1371/journal.pone.0210756 (PMC6336274; doi:10.1371/journal.pone.0210756)
Supplement: S2 Table — (DOCX) [file pone.0210756.s002.docx]

**Supporting Information**

Migratory patterns and settlement areas revealed by remote sensing in an endangered intra-African migrant, the Black Harrier *(Circus maurus);*

Marie-Sophie Garcia-Heras*^1,2*^*, Beatriz Arroyo^3^*^#^*, François Mougeot*^3#^*, Keith Bildstein*^4^*, Jean-François Therrien*^4^* & Robert E. Simmons^2#^

**S2 Table. More detailed summary data for the identified breeding and non-breeding settlement areas used by the 13 adult Black Harriers marked with GPS-GSM or PTT tracker devices** **followed in south-western South Africa during the 2008-2016** **period.**

| Name | Device | Period of year | Location | Number of locations | Number of days spent | Number of daily locations | 90% home range (km^2^) |
| --- | --- | --- | --- | --- | --- | --- | --- |
| F1 (orange) | PTT | Breeding2013, NA | Vanrhynsdorp, NC | 36 | 34 | 1.06 | 127.64 |
|  |  | Settlement1Y1 | Seymore, EC | 156 | 165 | 0.95 | 202.97 |
|  |  | Settlement2Y1 | Umgwali, EC | 40 | 48 | 0.83 | 77.67 |
|  |  | No Breeding2014 | Vanrhynsdorp, NC | 61 | 50 | 1.22 | 603.09 |
|  |  | Settlement1Y2 | Seymore, EC | 46 | 40 | 1.15 | 760.51* |
| F2 (light blue) | PTT | Breeding2012, failed | WCNP, WC | 21 | 7 | 3.00 | 45.62 |
|  |  | Settlement1Y1 | Overberg, WC | 276 | 148 | 1.86 | 65.31 |
|  |  | Breeding2013, successful | Nieuwoudtville, NC | 150 | 122 | 1.23 | 143.17 |
|  |  | Settlement1Y2 | Overberg, WC | 183 | 157 | 1.17 | 63.59 |
| F3 (light green) | GPS | Breeding2013, successful | Calvinia, NC | 1248 | 93 | 13.42 | 61.88 |
|  |  | Settlement1Y1 | Calvinia, NC | 262 | 32 | 8.19 | 14.88 |
|  |  | Settlement2Y1 | Sinxondo, EC | 208 | 25 | 8.32 | 18.32 |
|  |  | Settlement3Y1 | Daggakraal, MP | 545 | 108 | 5.05 | 21.07 |
|  |  | NoBreeding2014 | Vanrhynsdorp, NC | 612 | 115 | 5.32 | 43.78 |
| F4 (pink) | GPS | Breeding2014, successful | WCNP, WC | 482 | 67 | 7.19 | 5.39 |
|  |  | Settlement1Y1 | WCNP, WC | 367 | 83 | 4.42 | 7.94 |
|  |  | Settlement2Y1 | Strand, WC | 701 | 117 | 5.99 | 233.87* |
| F5 (dark grey) | PTT | Breeding2010, successful | WCNP, WC | 126 | 79 | 1.59 | 132.44 |
|  |  | Settlement1Y1 | Mokhotlong area, Lesotho, | 73 | 57 | 1.28 | 93.86 |
|  |  | Settlement2Y1 | Ngcobo area, EC | 53 | 89 | 0.60 | 320.16 |
|  |  | Breeding2011, successful | Cambeboo Mountain, EC | 150 | 148 | 1.01 | 261.89 |
|  |  | Settlement1Y2 | Mokhotlong, Lesotho | 118 | 75 | 1.57 | 91.85 |
|  |  | Settlement2Y2 | Ngcobo, EC | 20 | 36 | 0.56 | 184.27 |
|  |  | No Breeding2012 | Kambeboo Mountain, EC | 21 | 147 | 0.14 | 91.47 |
|  |  | Settlement1Y3 | Mokhotlong area, Lesotho, | 10 | 37 | 0.27 | 61.27 |
| F6 (yellow) | GPS | Breeding2015, failed | Jakkalsfontein, WC | 116 | 10 | 11.60 | 18.87 |
|  |  | Settlement1Y1 | WCNP, WC | 110 | 12 | 9.17 | 239.01 |
|  |  | Settlement2Y1 | Aberdeen, EC | 33 | 11 | 3.00 | 6.90 |
| F7 (brown) | GPS | Breeding 2013, successful | Vanrhynsdorp, NC | 1311 | 64 | 20.48 | 23.36 |
|  |  | Settlement 1Y1 | Stutterheim, EC | 1620 | 206 | 7.86 | 270.45* |
|  |  | No Breeding2014 | Veddrif, WC | 374 | 86 | 4.35 | 17.02 |
|  |  | Settlement 1Y2 | Stutterheim, EC | 22 | 52 | 0.42 | 167.32 |
| F8 (fuchsia) | GPS | Breeding2014, NA | Yzserfontein, WC | 739 | 72 | 10.26 | 21.11 |
|  |  | Settlement1Y1 | Tlokoeng, Lesotho | 361 | 103 | 3.50 | 74.69 |
|  |  | Settlement2Y1 | Klipfontein, MP | 566 | 94 | 6.02 | 106.49 |
|  |  | NoBreeding2015 | Yzerfontein, WC | 88 | 15 | 5.87 | 39.92 |
| F9 (black) | PTT | Breeding2008, NA | Koeberg , WC | 27 | 40 | 0.68 | 122.55 |
|  |  | Settlement1Y1 | WCNP, WC | 13 | 59 | 0.22 | 73.06 |
|  |  | Settlement2Y1 | Koeberg, WC | 11 | 83 | 0.13 | 23.26 |
| M1 (red) | PTT | Breeding2013, NA | Vanrhynsdorp, NC | 68 | 64 | 1.06 | 106.06 |
|  |  | Settlement1Y1 | East Graaff-Reinet, EC | 83 | 83 | 1.00 | 110.75 |
|  |  | Settlement2Y1 | West Graaff-Reinet, EC | 25 | 12 | 2.08 | 260.23 |
|  |  | No Breeding2014 | Vanrhynsdorp, NC | 88 | 20 | 0.22 | 148.41 |
| M2 (dark blue) | GPS | Breeding2013, successful | Nieuwoudtville, NC | 1104 | 63 | 17.52 | 110.53 |
|  |  | Settlement1Y1 | Numolani, Lesotho | 390 | 48 | 8.13 | 8.022 |
| M3 (dark green) | PTT | Breeding2013, successful | Calvinia, NC | 63 | 47 | 1.34 | 115.00 |
|  |  | Settlement1Y1 | Seymore, EC | 87 | 83 | 1.05 | 809.46* |
|  |  | Settlement2Y1 | Emandabeni, KZN | 60 | 68 | 0.88 | 164.30 |
|  |  | Settlement3Y1 | Mooi River, KZN | 25 | 34 | 0.74 | 33.69 |
|  |  | No Breeding2014 | Overberg, WC | 115 | 106 | 1.08 | 91.16 |
| M4 (turquoise) | PTT | Breeding2008, NA | WCNP, WC | 93 | 87 | 1.07 | 94.83 |
|  |  | Settlement1Y1 | WCNP, WC | 220 | 200 | 1.1 | 336.58 |

*individuals that used 2-3 locations within that specific settlement area

EC: Eastern Cape Province; NC: Northern Cape Province; WC: Western Cape Province; KZN: Kwazulu Natal; MP: Mpumalanga; WCNP: West Coast National Park

Each settlement area used by the birds during its annual movements is indicated, as well as the period of when the settlement was used. Adult females: F1-F9, adult males: M1-M4. The 90% home range sizes of each settlement area are also indicated.
